# Spreading of disturbances in realistic models of transmission grids in dependence on topology, inertia and heterogeneity

**DOI:** 10.1038/s41598-021-02758-2

**Published:** 2021-12-09

**Authors:** Kosisochukwu P. Nnoli, Stefan Kettemann

**Affiliations:** 1 Department of Computer Science and Electrical Engineering, Jacobs University Bremen, 28759 Bremen, Germany; 2 Department of Physics, Jacobs University Bremen, 28759 Bremen, Germany; 3 Division of Advanced Materials Science, Postech, Pohang, 790-784 South Korea

**Keywords:** Energy infrastructure, Energy grids and networks

## Abstract

The energy transition towards more renewable energy resources (RER) profoundly affects the frequency dynamics and stability of electrical power networks. Here, we investigate systematically the effect of reduced grid inertia, due to an increase in the magnitude of RER, its heterogeneous distribution and the grid topology on the propagation of disturbances in realistic power grid models. These studies are conducted with the DigSILENT PowerFactory software. By changing the power generation at one central bus in each grid at a specific time, we record the resulting frequency transients at all buses. Plotting the time of arrival (ToA) of the disturbance at each bus versus the distance from the disturbance, we analyse its propagation throughout the grid. While the ToAs are found to be distributed, we confirm a tendency that the ToA increases with geodesic distance linearly. Thereby, we can measure an average velocity of propagation by fitting the data with a ballistic equation. This velocity is found to decay with increasing inertia. Characterising each grid by its meshedness coefficient, we find that the distribution of the ToAs depends in more meshed grids less strongly on the grid inertia. In order to take into account the inhomogeneous distribution of inertia, we introduce an effective distance $r_{\mathrm{eff}}$, which is weighted with a factor which strongly depends on local inertia. We find that this effective distance is more strongly correlated with the ToAs, for all grids. This is confirmed quantitatively by obtaining a larger Pearson correlation coefficient between ToA and $r_{\mathrm{eff}}$ than with *r*. Remarkably, a ballistic equation for the ToA with a velocity, as derived from the swing equation, provides a strict lower bound for all effective distances $r_{\mathrm{eff}}$ in all power grids. thereby yielding a reliable estimate for the smallest time a disturbance needs to propagate that distance as function of system parameters, in particular inertia. We thereby conclude that in the analysis of contingencies of power grids it may be advisable that system designers and operators use the effective distance $r_{\mathrm{eff}}$, taking into account inhomogeneous distribution of inertia as introduced in Eq. (12), to locate a disturbance. Moreover, our results provide evidence for the importance of the network topology as quantified by the meshedness coefficient $\beta$.

## Introduction

The inertia of rotating masses of synchronized generators automatically stores kinetic energy which is immediately available in case of disturbances to stabilise the system. As a consequence of the energy transition to RER the inertia will decrease continuously. This may severely weaken the resilience of the grids against disturbances. The current energy market design does not provide sufficient incentives for such important system stabilising technologies^1^. The reason behind that is historical, since conventional power plants provide part of the necessary grid service automatically whenever power is generated. Therefore, it is necessary to find out how the resilience of the system can be guaranteed, in future. This necessitates a better understanding of future power systems, which we aim to contribute to, by studying the response of the transmission grid to disturbances under these new conditions.

The power system response to a disturbance is understood to unfold in four stages^2^: 1. electromagnetic stage, 2. inertial stage, 3. governor-response and 4. Automatic Generation Control (AGC) stage. The first stage begins the transient process at the time of disturbance. The response of the power system to this transient depends on the second and third stages describing the inertia and energy contributed by the spinning mass of synchronous generators as well as the primary response through the turbine-governor droop. The aftermath of these stages determines if the response is going to require the AGC stage response. This means, that in order to predict the time scales within which control measures would have to be in place after a contingency, it is crucial to understand the response to disturbances within the first two stages, and to get a better understanding of the time which disturbances need to spread a certain distance in a power grid in the presence of inertia. Moreover, as has been pointed out in Refs.^3,4^, there are multiple delays in the power system control, which can lead to power instabilities. Also the damping of interarea oscillations requires new type of control systems^5^. Therefore, proper control action requires an understanding of all time scales in the power grid and its dependence on the location of the contingency.

One way to achieve this is to extract the time which a disturbance needs to propagate in real power grids from direct frequency observations of power grids, as obtained from synchronized phasor unit measurements^6,7^. This has been achieved by the GridEye project as outlined in Ref.^8^ and by the gridradar project^9^. Such Wide-area monitoring systems (WAMS), using the real-time, global positioning system (GPS) time-synchronized measurements will be crucial to understand and control future power grids^10–12^, and are currently being set up in an increasing number of locations.

The other way to get a better understanding is, to study the spreading of disturbances in models of dynamic power grids. In Ref.^13^ a continuum approximation has been employed to derive an electromechanical wave equation, which for homogeneous parameters has the solution of decaying plain waves, propagating with a velocity *v*. This allows the derivation of the parametric dependence of the velocity *v*. This continuum electromechanical wave equation has been extended to allow for inhomogeneous parameters^14^. Another approach is to simplify the grid topology to a chain^13,15^. Other topologies of simplified network models based on second order swing equations have been considered in Refs.^16–18^.

As a measure of the disturbance spreading, the time of arrival (ToA) $t - t_0$ has been considered. The ToA with $t>t_0$ is defined to be the time, which a disturbance needs to arrive at distance *r* from the position of the disturbance which occurred at time $t_0$. It was found in Ref.^16^ analytically and in numerical simulations^17^ that for meshed grids and sufficiently large inertia a definite lower bound for the ToA at distance *r* is provided by a ballistic propagation formula, as given by $t-t_0 = r/v$, even though the ToAs at a distance *r* can be widely distributed. For homogeneous regular grids, the velocity *v* has been derived^13,16,17^ and its functional dependence on the power capacity of transmission lines, the inertia and the nominal grid frequency is known there, as outlined in the section on transient dynamic analysis. For low inertia and for tree-like grid topologies for any inertia, it was found in^17^ that the spreading changes qualitatively to a diffusive form, so that the lower bound for the arrival times depends quadratically on distance *r*. Inhomogeneities in the power grid typically result in anisotropic propagation and a wider distribution of ToAs. Moroever, disturbances may become localised in inhomogeneous grids^16^. The patterns of spreading in models of inhomogeneous power grids have been studied in Ref.^19^.

The aim of the present article is to study the spreading of disturbances in realistic transmission grid models systematically as function of system parameters, such as inertia, and in dependence on grid topology, by exploring a wide variety of grid models. In particular we consider the Nigerian 330 kV transmission network, the Ghanaian 161 kV transmission network as well as the IEEE 118 bus test- 138 kV grid. In addition, we study model grids with a Cayley-tree topology, and a Square-Grid topology, where the substations are modeled with the same parameters as the Nigerian 330 kV transmission network.

In the following sections, we will do the following: (1) introduce the five case study grids and their properties, (2) review the dynamic models of the power grids and their control devices, (3) review the load flow calculations and network stability analysis, (4) define the disturbance and analyse the electromechanical transient dynamics caused by it, (5) present the results of the simulations for the time of arrivals (ToA) of the disturbance at all buses analysed by fitting with a ballistic equation, extracting an averaged velocity, (6) introduce a weighted effective distances which takes into account the nonuniform distribution of inertia and is found to be strongly correlated with the ToAs, and (7) conclude in the last section with a summary of the main results and their relevance to the control design of future power grids.

## Description, modeling and simulation of electrical power networks

Let us start with the basic description of the five different network models used in the frequency dynamics investigations.

### Nigerian transmission grid model

**Figure 1:**
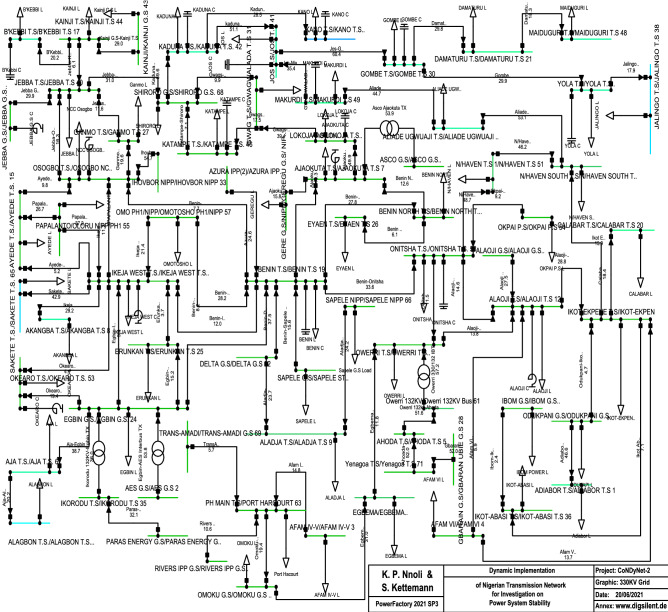
Single-line diagram of Nigerian 330 kV transmission network.

The Nigerian 330 kV transmission network comprises a total of 71 buses (substations/power terminals), 81 overhead transmission lines made from an alloy of aluminium and steel (with a limiting current of 1320 A) of which some consist of 2 or 3 circuit pairs. There are 107 less-decommissioned Unit generators which form the existing 29 power plant stations with a total power capacity of about 13, 208*MW*. The other voltage infrastructures include 132 kV, 66 kV (specifically for industrial utilities) and 33 kV sub-transmission networks. There are also 11 kV and 0.415 kV 3-phase distribution networks (for home and office utility areas). This network is of ring topology where many nodes are connected to one another in such a way that they form a closed loop prior to step-down^20^, as seen in the single line diagram shown in Fig. 1. All 330 kV voltage level buses, generators, controllers, and shunt compensators are represented^21^.

**Figure 2:**
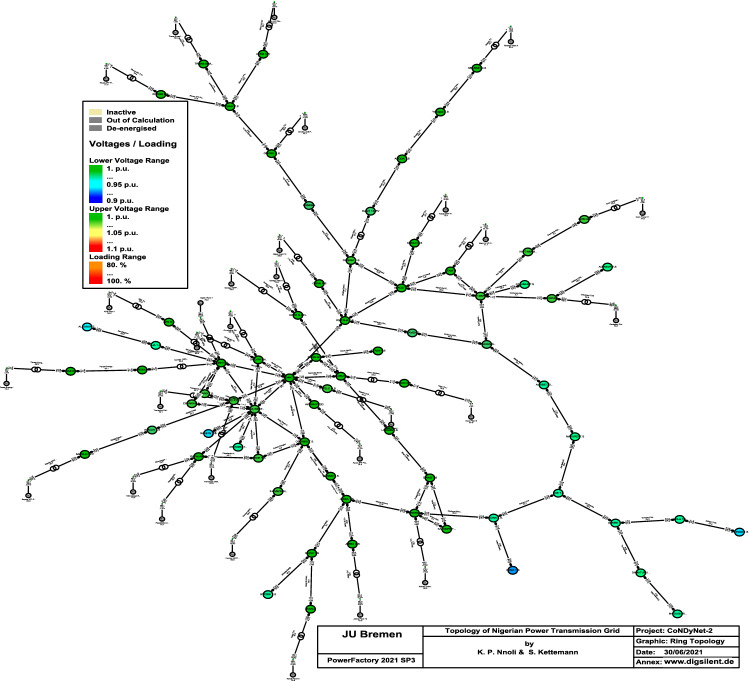
The topology of the reduced Nigerian 330 kV transmission network with $N_S =71$ buses.

For the purpose of simplifying the grid with respect to the number of generating units and to allow a comparison with other grid topologies without loosing their technical details and implications, we use in the following a reduced Nigerian 330 kV transmission network comprising of the 29 power plant stations with $N_S=71$ buses with their individual units integrated, as shown in Fig. 2 and listed and enumerated in the supplementary material^22^.

### Ghana transmission grid model

**Figure 3:**
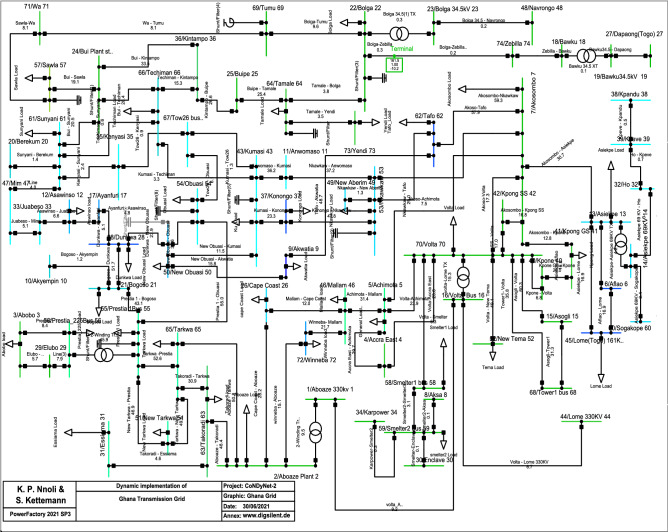
Single-line diagram of Ghana transmission network.

**Figure 4:**
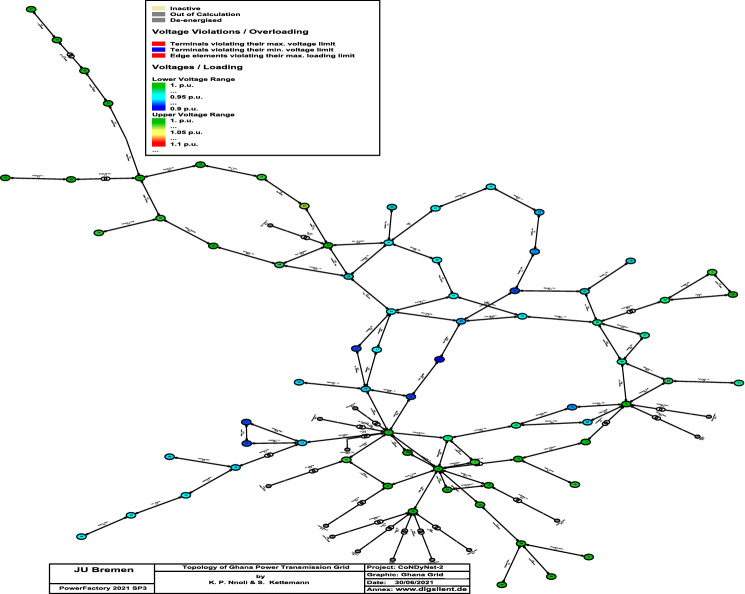
Ghana transmission network topology with N_S_ = 75.

Building on the results of^23^, with recent developments in the Volta River Authority that maintains the power systems in Ghana, the Ghana transmission grid comprises mostly of 161 kV high voltage transmission lines with only a few 330 kV and 225 kV line injections from neighbouring countries. Currently, its total power capacity is approximately 4888*MW* with about 90 overhead transmission lines connecting 74 buses^24^. The Ghana transmission network comprises about 7 hydro-powered plants and 8 gas-powered plants. The single line diagram and the topology of Ghana transmission grid are shown in Figs. 3 and 4, respectively.

### The IEEE 118 bus test-grid model and further grids

We also consider the IEEE 118-bus test-grid which depicts an approximation of the American Electric Power system in the U.S. Midwest as of December 1962^22^.

In order to enable a comparative study with power grids of different topology, we also study a Cayley-tree topology and a Square grid topology, where the substations are modeled with the same parameters as the Nigerian 330 kV transmission network and we choose the number of substations to be comparable, see the supplementary material^22^ for details.

As a quantitative measure of the topology of the different power grids, we introduce the meshedness coefficient $\beta$. It is defined by the ratio of the bounded faces of the buses using Euler characteristics and the maximum possible bounded faces in a given electrical network as^25^,

1 $$\begin{aligned} \beta = \frac{N_{\mathrm{LK}} - N_S + 1}{2N_S-5}, \end{aligned}$$

where $N_{\mathrm{LK}}$ and $N_S$ are the total number of overhead transmission lines plus inter-bus transformer links and buses, respectively, as listed in Table 1. Note that $\beta$ takes values between 0 and 1. It is weakly dependent on grid size $N_s$, but strongly dependent on grid topology: Tree grids are characterized by $\beta =0$, while $\beta =1$ indicates a completely meshed grid.

We use the following transmission line parameters in the modeling of these power grid: the line connections (i.e. from terminal i to terminal j), number of circuits, lengths (in km), positive and zero sequence resistance, *R*1 and *R*0, reactances *X*1 and *X*0, capacitances *C*1 and *C*0, susceptances *B*1 and *B*0 and rated current $I_{\mathrm{rated}}$. Included are also their respective synchronous machines and/ condensers describing their active *P*, reactive *Q*, and apparent *S* powers and power factor (pf)^20,21,23,24,26^. All data of the various grids used in this analysis are comparatively summarised in Table 1.

**Table 1:** Grid parameter values.

Grid data	Nigerian	Square	Cayley	Ghana	IEEE
Total active $P_{T}$ (MW)	13,208	13,208	13,208	4888	4374
Total rated $S_{T}$ (MVA)	15,450	15,450	15,450	5525	5112
Grid voltage V (KV)	330	330	330	161	138
Number of lines $N_L$	81	112	52	90	169
Number of links $N_{\mathrm{LK}}$	85	112	52	95	179
Meshedness coefficient $\beta$	0.11	0.40	0.00	0.15	0.27
Avg. Line-length a (Km)	92	100	100	59	46
Number of PV buses $N_{\mathrm{PV}}$	25	22	21	7	19
Number of gen stations $N_G$	29	29	29	14	19
Number of substations/buses $N_S$	71	64	53	74	118
Total number of effective buses $N_{\mathrm{r_{eff}}}$	25	22	21	7	54
Avg. Line-inductance L per length (mH/Km)	1.076	1.054	1.054	1.332	1.292

## Dynamic model for power grids and control devices

The swing equation describes the balance between the mechanical turbine torque $T_t$ in N.m. of the plant’s individual turbines and the electromagnetic torque $T_e$ in N.m. of their synchronous generators, as governed by the differential equation^27–29^,

2 $$\begin{aligned} J_i\frac{d\omega ^R_{i}}{dt} + D_{wi}\omega ^R_{i} = T_t - T_e, \end{aligned}$$

where $D_{wi}$ accounts for rotational loss due to windings for the generator at bus *i* in N.m.s. Here *i* denotes the index of power generators in the grid, *t* time in seconds, $J_i = 2 H_i S_{i}/ \omega ^{2}_0$ is the combined moment of inertia of the turbine and generator in kg   $\hbox {m}^2$ with $H_i$ the generator inertia constant in seconds and $S_{i}$ the generator apparent power in volt-amperes (VA). Also, $\omega ^R_{i}$ is the angular velocity of the rotor in electrical rad/s and $\omega _0$ is its rated synchronous value in electrical rad/s. We assume that any change in the rotor’s angular velocity is a derivative of its angular position $\delta$ (in electrical radians with respect to its synchronously rotating setpoint $\delta _0$), given by $\omega ^R_{i}-\omega _0 = d\delta _i/dt.$ Taking the derivative of $\omega ^R_{i}$ with respect to time, we get, since $\omega _0$ is constant,

3 $$\begin{aligned} \frac{d\omega ^R_{i}}{dt} = \frac{d^2\delta }{dt^2}. \end{aligned}$$

Here $\omega _0 = 2\pi v_o$ with the nominal grid frequency $v_o$ which is 50 or 60 Hz depending on a country’s grid code. Substituting Eq. (3) into Eq. (2), we find

4 $$\begin{aligned} J_i\frac{d^2\delta _i}{dt^2} + D_{wi}\Big (\frac{d\delta _i}{dt}\Big ) = T_m - T_e. \end{aligned}$$

Here, the net mechanical shaft torque $T_m=T_t-D_{wi} \omega _0$, which is the turbine torque minus the rotational losses at $\omega _0$ is introduced^29^. Multiplying both sides of Eq. (4) by the rated speed $\omega _0$ we get, since power is a product of torque and angular velocity,

5 $$\begin{aligned} J_i\omega _0\frac{d^2\delta _i}{dt^2} + D_{wi}\omega _0\Big (\frac{d\delta _i}{dt}\Big ) = P_m - P_e, \end{aligned}$$

where $P_m$ is the mechanical power from the turbine and $P_e$ is the electrical power from the generator’s air-gap. If we denote the angular momentum of the rotor at rated speed with $M_i$ (i.e. $M_i = J_i\omega _0 = \frac{2H_i}{\omega _0}S_{i}$) and also denote the damping coefficient at rated synchronous speed with $D_i$ (i.e. $D_i= D_{wi}\omega _0$), the swing equations can be written as^30–32^,

6 $$\begin{aligned} \frac{2H_i}{\omega _o}S_{i}\frac{d^2\delta _i}{dt^2} + D_i\frac{d\delta _i}{dt} = P_i + \sum _{j=1}^{N_S}K_{ij} \sin (\delta _j-\delta _i). \end{aligned}$$

with the number of nodes $N_S$, the power in node *i*,  $P_i$ and the transmission line power capacity between *i* and *j*, $K_{ij}$.

The devices used to control the outputs of the synchronous generating-units in all model grids include, but are not limited to: the automatic voltage regulator (AVR), responsible for voltage control of the generator through its excitation control; the speed governor used in the model is the turbine governor (TGOV-model) of the generator’s prime mover which is responsible for keeping the generator’s rotor frequency synchronous to the grid frequency, thereby maintaining the frequency operational limits when there are contingencies. The control devices for the power plants also comprise the power system stabilizer (PSS), tasked with reducing the oscillatory instability and enhancing the damping of the power system oscillations through excitation control. The controlled signal is a derivative of the generator rotor speed injected to the AVR through the excitation system to cancel out the phase lag between exciter’s voltage set reference and the generator’s windings torque^33^. In a basic power plant frame, it is also important to add over-excitation limiter (OEL) and the under-excitation limiter (UEL) for the AVR device. Here, we carry out stability studies without the OEL and UEL devices, as many power plants stations may not have them.

The choice of the synchronous machine models are based on IEEE guide for synchronous generator modeling practice and applications in power system stability analysis^34^. The turbine governor models used in the design simulations were chosen according to IEEE recommendations for dynamic models of turbine-governor in power system studies^35^. The following two models were used for the Nigerian, Cayley-tree, Square and Ghanaian power networks and the synchronous condensers part of IEEE 118-bus network: 1. The HYGOV model was used for the hydro power plants. 2. The simplified TGOV1 model was used for the thermal plants. The operations and basic signals are the same in both models. The excitation voltages of the synchronous generators in the networks are controlled by the simplified excitation (SEX-AVR) model and serve as their excitation and voltage control system except for the part of the synchronous generators in the IEEE 118 bus test-grid where the primary IEEET1-AVR model was used.

The choice of the automatic voltage regulator for synchronous generators was influenced by ENTSO-E^36^. The PSS2A model was used for all power plants in the networks. Each was tuned to ensure that no destabilising signal is fed back into the excitation system voltage summation point. Power system stabilizers remain the most cost efficient way of enhancing the damping of power system oscillations. The PSS2A standard model used in this design was influenced by IEEE recommended practice for excitation system models for power system stability studies^37^.

## Load flow and electromechanical transient stability analysis in PowerFactory

Load flow calculation in complex power grids is used to determine voltage magnitude *V* and angle $\theta$ of the substations/buses, as well as active P and reactive Q power^27^. Here, DigSILENT PowerFactory is used to implement the Newton-Raphson (power equation, classical) method as its non-linear equation solver because of its fast-convergence. This method is used to obtain the initial conditions during the top of the maximum power flows in all power grids. The currents at steady state are calculated from $I_i=S_i^*/V_i= (P_i - jQ_i)/V_i$, where $i =$ 1, 2, ...; $N_G$ and *j* the imaginary unit with $N_G$ the total number of generators in the generating stations. The currents at the substations are given in terms of the admittance matrix by $I_{\mathrm{bus}}= Y_{\mathrm{bus}} V_{\mathrm{bus}}$. All dynamic components of the plants (AVR, PSS, Turbine / GOV models) and all system parameters are modeled and simulated. The active power balance is ensured by direct coupling of the grid frequency to synchronous generators, ensuring inertia activation and nodal relaxations. The phase coupling between generators is described by the swing equations with frequency-dependent load damping. The effect of increasing the injection of renewable energy resources on the stability of the electrical power networks is studied by varying the aggregated inertia constant $H_{\mathrm{grid}}$ of the entire grid by^38^,

7 $$\begin{aligned} H_{\mathrm{grid}} = \frac{\sum _{i=1}^{N_S}H_i S_i}{S_{T}}, \end{aligned}$$

with total rated apparent power in the grid $S_{T}=\sum _{i=1}^{N_S}S_i$, where $S_i$ and $H_i$ are rated power and inertia constant of the *i*th bus, respectively. Note that $H_i=0$ on non-generator buses.

The voltage profiles from stationary power flow were examined at no transient to ensure that the buses maintain the voltage tolerance according to the Nigerian grid code which specifies 0.85 p.u. minimum (i.e., 280.5 kV) and 1.05 p.u. maximum (i.e., 346.5 kV) tolerances. Voltage profiles of the Nigerian, Square, Cayley-tree, Ghana and IEEE 118 test power networks at steady operating conditions (undisturbed operation) were captured and represented in bar-charts in the supplementary material^22^. The results show that all buses were within the specified voltage tolerance. As overloading of power generators is defined to be any loading above 80$\%$ we choose this as our loading thresholds. The grid code specifies frequencies of 0.995 p.u. (min) and 1.005 (max) tolerance for network elements. Frequencies of 0.99 p.u. (min) and 1.01 p.u. (max) are used in actual daily operational procedure^39^.

**Figure 5:**
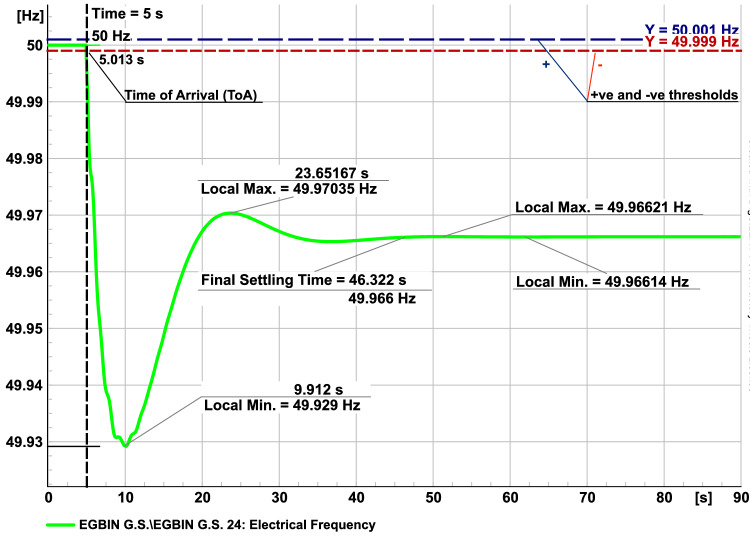
Green line: Frequency as a function of time at a bus where a synchronous machine (SM) Event occurs at $t_0= 5$ s. Blue line: Frequency deviation threshold at 50.001 Hz. Red Line: Deviation threshold at 49.999 Hz. Relevant times scales are indicated by thin lines: the Time of Arrival (ToA), the time when first local minimum is reached, the time when first local maximum is reached and the frequency settling time.

## Transient dynamic analysis

The magnitudes of the injected perturbations $P_{\mathrm{inj}}$ for the synchronous machine (SM) events are chosen in such a way to make the transient dynamic analysis in the various power grids comparable. Therefore, we choose the disturbance to be $P_{\mathrm{inj}}= 0.5 P_{T}/N_{ \mathrm PV}$, with $P_{T}= \sum _{i=1}^{N}P_i$ the total active power in the grid and $N_{\mathrm{PV}}$ the total number of generator buses participating in the power injections as given in Table 1. Here, a PV bus is a bus from which generator(s) are connected to the power system. A comparison chart of the power in *MW* that participated in the SM-Event is shown in Table 2. Outage contingencies were simulated at those power plants substations which have the minimum geodesic distances to the rest of the substations for each grid. A geodesic distance between two nodes in a network is defined to be the minimal number of edges which connect these two nodes in a given network. That way they are most centrally located and the spread of disturbances throughout the grid can be monitored.

**Table 2:** Injected-perturbations in the grids.

Perturbation $P_{ \mathrm inj}$	Nigerian	Square	Cayley-tree	Ghana	IEEE 118
SM-event (MW)	264	300	314	349	115

As shown in Fig. 5 the transient is characterised by several times scales, indicated by thin lines. We choose for the analysis the Time of Arrival (ToA), which measures the time, the frequency deviation first surpasses a threshold. We place the threshold meter at very small values of $\delta \nu =\pm$ 0.001 Hz, in order to record the propagation of the disturbances most directly. The synchronous machine event is chosen to occur at exactly $t_0= 5$ s in the 90 s transient electromechanical stability simulation time frame. The switching at the event takes a finite time of $\Delta t= 200$ ms. The IEEE 118 Bus test-grid has a nominal $\nu _0 = 60$ Hz frequency. All other grids have $\nu _0 = 50$ Hz as nominal frequency. In order to be able to focus the investigation of the transient on the first two stages, and in particular on the contribution of grid inertia on the disturbance spreading pattern and speed, we choose to turn off any controller in these numerical experiments.

## Disturbance propagation analysis

**Figure 6:**
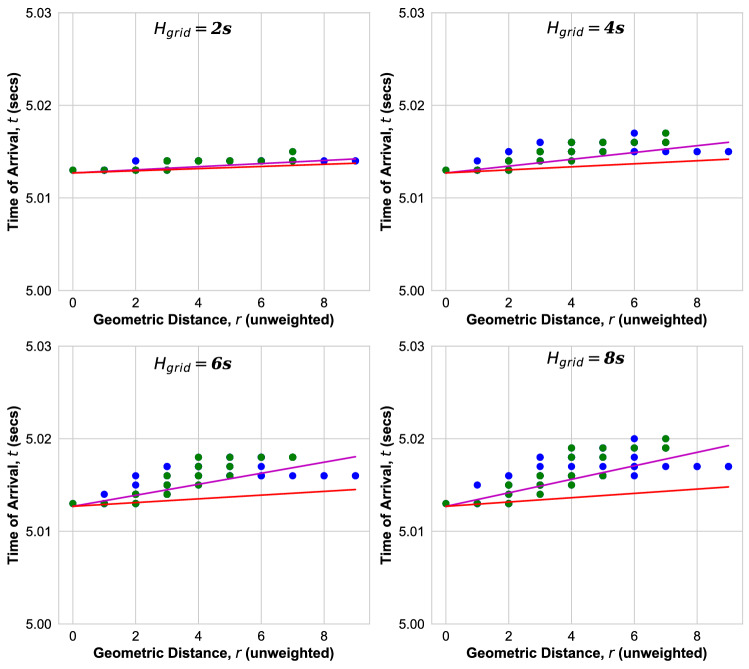
ToA versus geodesic distance *r* for Nigerian power grid from transient dynamic simulations for inertia $H_{\mathrm{grid}}$. Green (Blue) dots: ToA at bus(es) with at least one (no) inertia injection. Magenta line: fit to ballistic Eq. (8). Red line: ballistic Eq. (8) with velocity Eq. (10).

In the following, we show results of the measurement of the ToA at each bus after the event at time $t_0,$ as obtained from the PowerFactory simulation. In Fig. 6, the ToAs for all buses of the Nigerian grid are shown as function of their geodesic distance *r* from the event location. Results are obtained for different aggregated grid inertia of $H_{\mathrm{grid}}$ = 2s, 4s, 6s, 8s, as varied by multiplying all local inertia $H_i$ by a common factor.

We see that, while the ToAs at nodes with same geodesic distance *r* are distributed over some range, they tentatively increase with *r* from the event location, as expected. We observe that the width of the ToA distribution increases with inertia $H_{\mathrm{grid}}$ in the Nigerian grid. If the propagation would be ballistic, the ToAs would increase linearly with distance *r* as

8 $$\begin{aligned} t = b_0 +r/v, \end{aligned}$$

and one can identify the velocity *v* with which the perturbation spreads. We therefore fit all ToAs as function of *r* to the ballistic Eq. (8), magenta lines in Fig. 6, see supplementary material for further documentation^22^. The parameter $b_0$ is typically larger than $t_0= 5.0$ s due to the fact that the synchronous machine contingency may have a small delay and the switching takes a small time of order $\delta t = 200$ ms. In order to better understand these results, let us review the solution of the swing equation Eq. (6) for homogeneous parameters, where it can be solved analytically. For meshed grids and sufficiently large inertia one finds that a disturbance spreads ballistically according to Eq. (8) with velocity

9 $$\begin{aligned} v=\sqrt{\frac{K}{(\omega _0 J)}} a, \end{aligned}$$

where *a* is the length of a transmission line. Equation (9) can be derived by linearising the swing equation in phase deviations due to the disturbance, which yields a wave equation on the power grid network^13,16,17^. Here, *J* is the inertia of a synchronous generator or motor, respectively. In real power grids all parameters are inhomogeneous: The power capacity $K_{ij}$ and the length $a_{ij}$ are different for every transmission line between nodes *i* and *j* and the inertia is different for every generator, $J_i$ at node *i*, with some of the nodes having no inertia. Let us therefore introduce as a measure of the velocity in real grids, the effective velocity

10 $$\begin{aligned} v_{\mathrm{eff}} = \sqrt{\frac{{\bar{K}}\omega _0}{2 H_{\mathrm{grid}} S_B}} {\bar{a}}, \end{aligned}$$

which depends on the following averaged parameters: ${\bar{K}} = \sum _{i < j}K_{ij}/N_L,$ is the average power capacity of the $N_L$ transmission lines, where $K_{ij}= V^2/(\omega _0 L_{ij})$ is the power capacity of a purely inductive transmission line between nodes *i* and *j*, *V* the voltage and $L_{ij}$ the line positive sequence inductance. ${\bar{a}}$ is the average length of the transmission lines. $H_{\mathrm{grid}}$ is the aggregated inertia constant, Eq. (7). $S_B= S_T/N_S$ is the total rated grid power in units of *MVA* divided by the total number of buses $N_S$. With these definitions we are now ready to compare with the simulation results in realistic power grids with inhomogeneous parameters, plotting Eq. (8) (red line) with the velocity Eq. (10) in Fig. 6.

**Figure 7:**
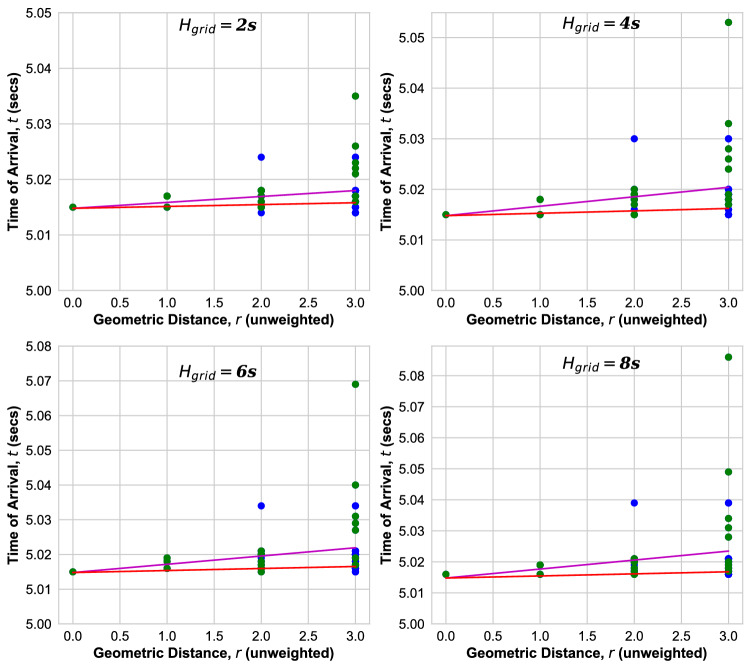
ToA versus *r* for the Cayley-tree Power grid as obtained for inertia $H_{\mathrm{grid}}$. Green (Blue) dots: ToA at bus(es) with at least one (no) inertia injection. Magenta line: fit to ballistic Eq. (8). Red line: ballistic Eq. (8) with velocity Eq. (10).

We observe for the Nigerian power grid in Fig. 6 that the ballistic Eq. (8) with the velocity calculated according to Eq. (10) (red line) provides a lower bound for the ToAs at all nodes and for all inertia $H_{\mathrm{grid}}$ = 2s, 4s,6s,8s. The measured ToAs are found to be distributed in an interval $b_0 +r/v_{\mathrm{eff}}< t < b_0 -r/v_{\mathrm{min}}$, whose width is found to increase with inertia. We observe that the minimal velocity $v_{\mathrm{min}}$ decreases with increasing inertia.

In order to understand the reason for this distribution, we plot the ToAs at buses with no inertia in Fig. 6 in blue, while the green dots indicate that at least one of the buses at this distance *r* and with this arrival time has finite inertia. From this we can conclude that one reason that the ToAs are widely distributed for same geodesic distance *r* could be the fact that the propagation is delayed in nodes with inertia while in nodes without inertia there is no delay. This can clearly be seen in Fig. 6, in particular for the example of the bus at distance $r=9$, where the disturbance arrives at the same time as at the node at distance $r=8$, which has no inertia.

For the Cayley-tree power grid we observe that the ToAs are more widely distributed, Fig. 7. Moreover, the ballistic Eq. (8) with velocity Eq. (10) no longer provides a lower bound for the ToAs at all buses. Rather, there are outliers, which are the ToAs falling below the red line. In the Square grid we observe that the ballistic Eq. (8) with the velocity calculated according to Eq. (10) does provide a lower bound for the ToA at all buses for the smallest inertia $H_{\mathrm{grid}}=2$ s, while there are outliers at higher $H_{\mathrm{grid}}$, as documented in Ref.^22^.

**Figure 8:**
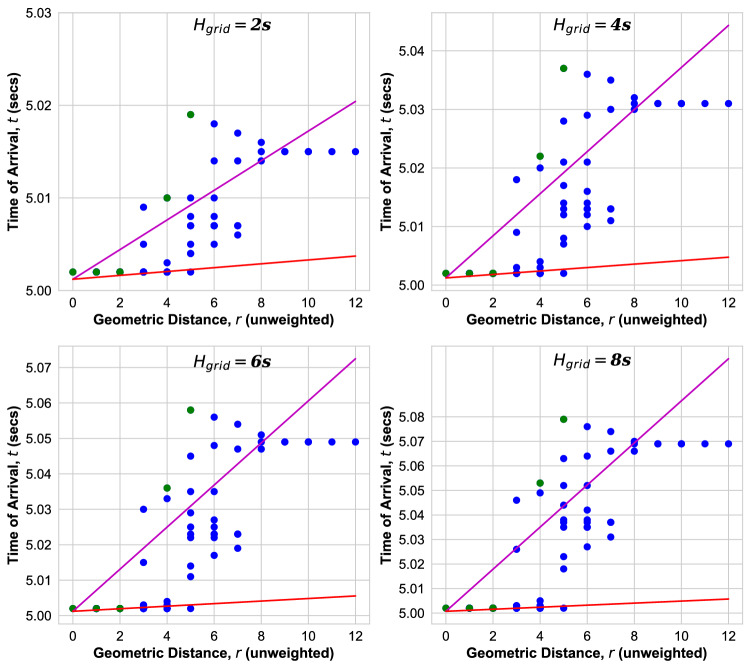
ToA versus *r* for Ghana Power grid as obtained from transient dynamic simulations for inertia $H_{\mathrm{grid}}$. Green (Blue) dots: ToA at bus(es) with at least one (no) inertia injection. Magenta line: fit to ballistic Eq. (8). Red line: ballistic Eq. (8) with velocity Eq. (10).

Figure 8 shows the ToA as function of *r* in the Ghana grid. The ballistic Eq. (8) with velocity calculated according to Eq. (10) (red line) provides a lower bound for the ToAs at small and large distance, while there are outliers for all $H_{\mathrm{grid}}$ at intermediate distances $r= 2 ,\ldots ,5$. In addition, we observe that there is a wide distribution of ToAs at intermediate distance *r* whose width increases with an increase in $H_{\mathrm{grid}}$.

**Figure 9:**
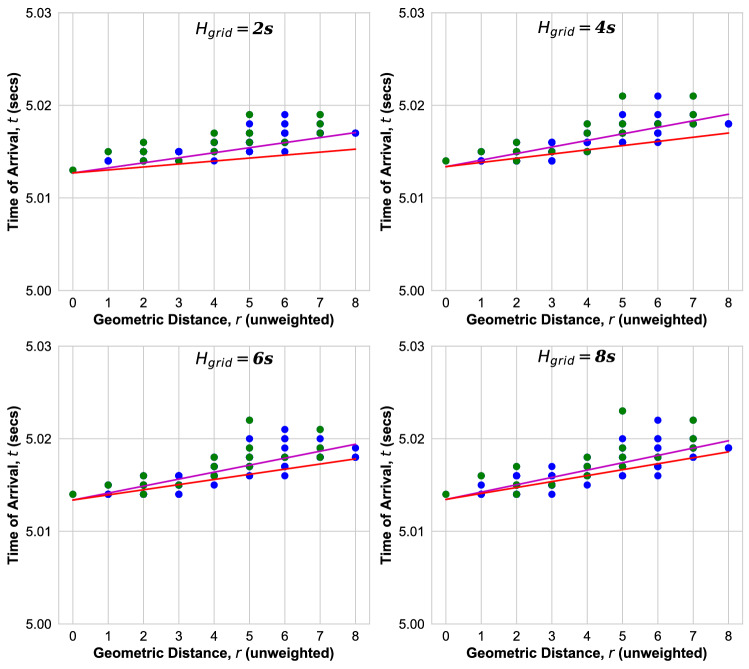
ToA versus *r* for IEEE Power grid as obtained from transient dynamic simulations for inertia $H_{\mathrm{grid}}$. Green (Blue) dots: ToA at bus(es) with at least one (no) inertia injection. Magenta line: fit to ballistic Eq. (8). Red line: ballistic Eq. (8) with velocity Eq. (10).

**Figure 10:**
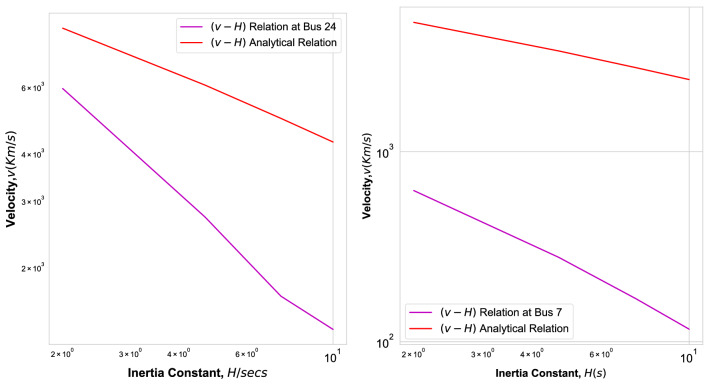
Fitted velocity *v* as function of inertia *H* (Magenta Line) and velocity Eq. (10) (red line) for Nigerian Power Grid Fig. 6 with contingency at bus 24 (left) and for Ghana Power Grid, Fig. 8 with contingency at bus 7 (right) in double logarithmic plot.

In the IEEE 118-bus test grid shown in Fig. 9, we observe that the ballistic equation with velocity Eq. (10) provides a lower bound for ToAs (red line) only for lowest inertia $H_{\mathrm{grid}}=2$ s. For higher inertia, there are outliers at intermediate *r* which are located at the nodes with no inertia. The distribution of ToAs is narrower than in the other grids. This may be because of the high degree of meshedness of this grid similar to what we observed in the square grid in Ref.^22^.

In Fig. 10 we plot the fitted (magenta) and analytical (red) velocity versus inertia *H* for the Nigerian (left) and the Ghana (right) power grid. We observe that the fitted velocity decays with the inertia faster than the analytically predicted power law $v \sim H^{-1/2}.$ We find for the IEEE 118-bus test grid as well as for the square grid that the fitted velocity decays with the inertia more slowly than the analytical velocity which decays with the power law $\sim H^{-1/2},$ as documented in Ref.^22^.

In order to understand the effect of grid topology on these results better, let us compare the degree of meshedness Eq. (1) as listed in Table 1 for the various grids. It is vanishing $\beta =0$ for the Cayley tree grid, and it is small, $\beta =0.11$, for both the Nigerian and for the Ghanaian grid, where it is $\beta =0.15$. We note that both the Nigerian and Ghana grid have a ring like topology at the center of the grid, while there are many tree like connections to outer buses, resulting overall in a small meshedness coefficient $\beta$. It is substantially larger in the IEEE test grid $\beta =0.27$, and largest (in the considered grids) in the square grid $\beta =0.4$.

From the results for the ToAs versus distance *r* we notice that the more meshed a power grid is, as quantified by a higher meshedness coefficient $\beta$, the weaker does the distribution of ToAs depend on the grid inertia $H_{\mathrm{grid}}$. This can also be seen from a weaker dependence of the fitted ballistic velocity on the inertia in both Square and IEEE 118-bus test grid.

Finally, we observe that in tree like grids with small $\beta$ there is a wide spread of ToA. In particular, there are buses where the disturbance arrives much later than expected in a meshed grid. This observation is in accordance with Ref.^17^, where it was found that a disturbance tends to be more easily localised in a tree like grid, preventing or at least slowing down its spreading. Thus, the response to the contingency is in some buses slower in less meshed grids, like the Cayley-tree and the Ghana grids, as compared to the more meshed grids like the Square grid and the IEEE grid^22^.

## Weighted effective distance

In order to account for the variation of inertia in the buses, let us introduce a properly weighted effective distance $r_{\mathrm{eff}}$. We begin by extending the analytical result for the velocity as derived from the swing equations to a velocity $v_{i j}$ of the disturbance when propagating between nodes *i* and *j* for inhomogeneous parameters,

11 $$\begin{aligned} v_{i j} = {\mathrm{min}} \left( \sqrt{\frac{K_{ij}}{J_i \omega }} a_{ij}, c_{ij} \right) , \end{aligned}$$

where $a_{ij}$ is the length of this transmission line. In a transmission line which has no or low Ohmic losses, the velocity $c_{ij}$ along the transmission line between nodes *i* and *j* can be derived from the telegraph equation as a function of its inductance and capacitance^40^ as $c_{ij}= 1/ \sqrt{L_{ij}C_{ij}}$, yielding a velocity which is close to the velocity of light $c= 3\times 10^8$ m/s for high voltage transmission lines. However, when there is inertia $J_i$ at node *i* the transmission is delayed slowing the velocity down to a value $v_{i j} = \sqrt{K_{ij}/(J_i \omega )} a_{ij}$. Only if there is no inertia in node *i*, $J_i =0$, the disturbance spreads to the next node faster, with velocity $c_{ij}$, close to the speed of light. The inhomogeneous distribution of inertia is thus one of the reasons that the observed ToAs are distributed widely, and that there are nodes which are reached by the disturbance faster than expected in a homogeneous grid. According to Eq. (11) we expect that nodes which are connected with the location of the disturbance through nodes with low or no inertia are reached faster by the disturbance. Let us therefore take this fact into account.

**Figure 11:**
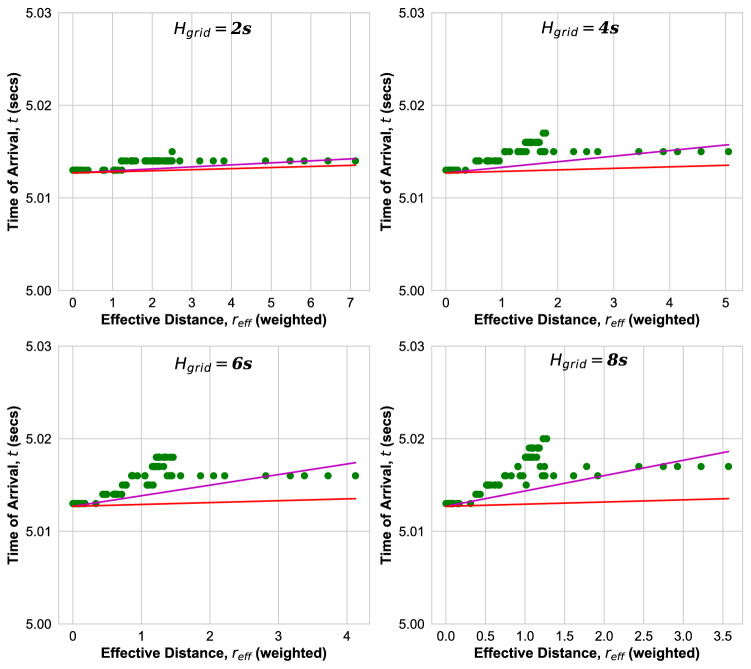
ToA versus effective distance for the Nigerian Power grid according to Eq. (12) for different grid parameter $H_{grid}$. Magenta line: fit to ballistic Eq. (8). Red line: ballistic Eq. (8) with velocity Eq. (11).

**Figure 12:**
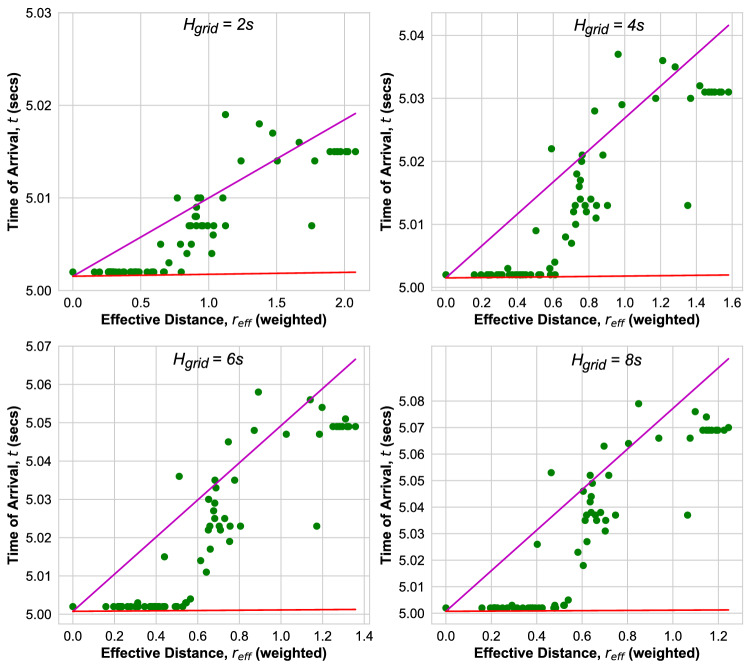
ToA versus effective distance for the Ghana Power grid according to Eq. (12) for different grid inertia $H_{\mathrm{grid}}$. Magenta line: fit to ballistic Eq. (8). Red line: ballistic Eq. (8) with velocity Eq. (11).

Using Eq. (11) a more accurate estimate for the ToA at a node *l* when the disturbance occurs at node *k* is given by $t_{kl} - b_0 = {\mathrm{min}}( \sum _{ij {\mathrm ~ along ~ Path~ from ~k ~ to~ l}} a_{ij}/v_{ij}),$ where the sum is over all pairs of nodes *i*, *j* along the path between nodes *k* and *l* which has the minimal arrival time. Accordingly, we define an effective distance between nodes *k* and *l* which is weighted with the inverse of the velocity of the disturbance between nodes *i* and *j*, Eq. (11),

12 $$\begin{aligned} r_{\mathrm{eff}}^{k l} = \frac{ v_{\mathrm{eff}} }{{\bar{a}}} {\mathrm{min}} \bigg ( \sum _{i,j {\mathrm{~ along ~ Path~ from ~k ~ to~ l}}} \frac{a_{ij}}{v_{ij} } \bigg ), \end{aligned}$$

where $v_{\mathrm{eff}}$ is the effective velocity defined in Eq. (10) and ${\bar{a}}$ the average transmission line length. We note that, since $v_{ij}$ strongly depends on the presence and magnitude of inertia at node *i*, this effective distance is weighted with a local factor which takes into account the amount of inertia present on the geodesic path between nodes *k* and *l*.

**Figure 13:**
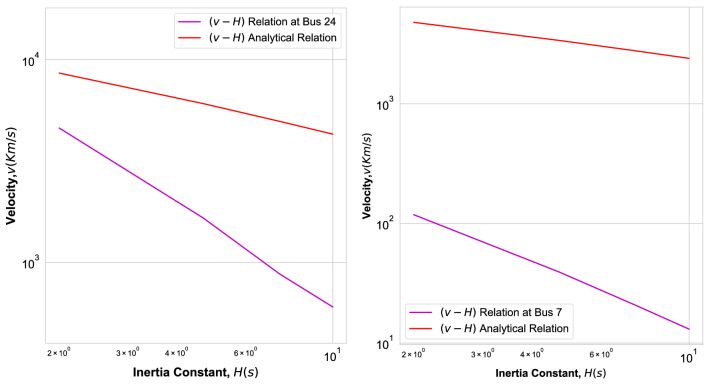
Fitted velocity *v* (Magenta) and velocity Eq. (10) (red) when the ToA is plotted versus effective distance for the Nigerian (left) and the Ghana Power Grid as function of inertia parameter *H* in double logarithmic plot.

Now, let us plot the measured ToAs versus this effective distance $r_{\mathrm{eff}}$, in order to see if we thereby get a less scattered plot and fewer outliers. We consider in particular those grids where we observed many outliers, namely the Nigerian, Cayley-tree and Ghana power grid. In Fig. 11 we plot the times of arrival versus $r_{\mathrm{eff}}$ for the Nigerian grid. First of all, we observe that the ballistic motion with the analytical velocity Eq. (10) provides now a strict lower bound for all effective distances (red line). Secondly, the ToAs are less widely distributed when compared to the plot versus geodesic distances in Fig. 6. For the Cayley tree grid we also find that the ballistic motion with the analytical velocity Eq. (10) provides now a strict lower bound for all ToAs when plotted as function of effective distance^22^, but its ToAs remain distributed without a strong correlation with the effective distance $r_{\mathrm{eff}}$. Figure 12 shows the ToAs as function of effective distance in the Ghana grid. There are no outliers and in comparison with Fig. 8, the width of the distribution of ToAs decreased strongly, the ToAs monotonuosly increase with $r_{\mathrm{eff}}$ with few exceptions. Figure 13 shows a plot of the fitted ballistic velocity (magenta) and the analytical velocity (red) versus inertia constant $H_{\mathrm{grid}}$ for the Nigerian power grid (left) and the Ghana grid (right) as obtained by fitting all ToAs as function of effective distance $r_{\mathrm{eff}}$. It monotonously decays as a power law with inertia in both grids, faster than the analytical results t$v \sim H^{-1/2}$.

To statistically quantify the correlation of the frequency ToAs with the geodesic distance from the contingency *r* and effective distance $\hbox {r}_{\mathrm{eff}}$ for the various power grids, we calculated the Pearson correlation coefficient $C_r$ between all ToAs and *r* as given by

13 $$\begin{aligned} C_r= \frac{\sum ^{N_S}_{i}(\mathrm ToA_i - \overline{\mathrm{ToA}}) (r_i - {\overline{r}})}{\sqrt{ \sum ^{N_S}_{i}(\mathrm ToA_i - \overline{\mathrm{ToA}})^2 \sum ^{N_S}_{i}(r_i - {\overline{r}})^2}} \end{aligned}$$

and accordingly the Pearson correlation coefficient $C_{r\mathrm eff}$ between all ToAs and their respective $r_{\mathrm{eff}}$ as given by

14 $$\begin{aligned} C_{r\mathrm eff}= \frac{\sum ^{N_S}_{i}(\mathrm ToA_i - \overline{\mathrm{ToA}}) (r_{\mathrm{eff} i} - {\overline{r}}_{\mathrm{eff}})}{\sqrt{ \sum ^{N_S}_{i}(\mathrm ToA_i - \overline{\mathrm{ToA}})^2 \sum ^{N_S}_{i}(r_{\mathrm{eff} i} - {\overline{r}}_{\mathrm{eff}})^2}} , \end{aligned}$$

where $ToA_i$ is the measured time of arrival at node *i* at geodesic distance $r_i$ and effective distance $r_{\mathrm{eff} i}$. $\overline{\mathrm{ToA}}$, ${\overline{r}}$ and ${\overline{r}}_{\mathrm{eff}}$ are the respective mean values. The *C*s for each grid inertia (i.e., $H_{\mathrm{grid}}$) are shown in Table 3. We observe that $C_{r\mathrm eff}$ is larger than $C_r$ for all power grids and all inertia values $H_{\mathrm{grid}}$. Thus, the effective distance r$_{\mathrm{eff}}$ correlates more strongly with the ToAs than the geodesic distance *r*, confirming our observations above when plotting ToA versus *r* and r$_{\mathrm{eff}}$. Also, we observe that the smaller the meshedness coefficient $\beta$ of the power grid is, Table 1, (Ghana $\beta =0.15$, Nigerian $\beta =0.11$, Cayley tree $\beta =0$), the smaller both correlation coefficients are. While $C_r$ depends for all power grids rather strongly on the inertia value $H_{\mathrm{grid}}$, the correlation with $r_{\mathrm{eff}}$, $C_{r\mathrm eff}$ depends only weakly on it in the Nigerian and Ghanaian power grid.

**Table 3:** Pearson correlation coefficients.

Grid	*C*	ToA$_{\mathrm{H=2s}}$	ToA$_{\mathrm{H=4s}}$	ToA$_{\mathrm{H=6s}}$	ToA$_{\mathrm{H=8s}}$
Nigerian	$C_r$	0.712	0.692	0.740	0.741
	$C_{r\mathrm eff}$	0.768	0.767	0.793	0.769
Ghana	$C_r$	0.810	0.812	0.820	0.829
	$C_{r\mathrm eff}$	0.885	0.896	0.899	0.899
Cayley-tree	$C_r$	0.211	0.213	0.198	0.184
	$C_{r\mathrm eff}$	0.218	0.295	0.303	0.311

We note that another inertia weighted distance was introduced previously in Ref.^41^ which was used in Ref.^42^ to suggest that electrically-interfaced control resources enabled with damping should be placed at locations further away from the center of inertia (COI). That inertia weighted electrical distance is thereby rather different than the one we introduced here, $r_{\mathrm{eff}}$, Eq. (12), which not only takes into account the inhomogeneous inertia, but also the inhomogeneous transmission line length $a_{ij}$ and power capacity $K_{ij}$.

## Summary and conclusions

The spread of a disturbance in transmission power grids was studied on a large variety of realistic network models by means of the DigSILENT PowerFactory software. We measured the time of arrival (ToA) of a disturbance and analysed it systematically as function of the distance of the buses to the contingency. By plotting the measured ToA versus the geodesic distance *r* from the location of the perturbation, we find only a weak correlation between these measures. Lowering the inertia in the grid is found to reduce the ToA irrespective of the grid topology. Thus, a disturbance travels faster with less grid inertia, or equivalently, with more RES in the grid. Characterising each grid by its meshedness coefficient, we find that the distribution of the ToAs is in more meshed grids only weakly affected by a change in grid inertia. Moreover, we observe that in tree like grids, like the Cayley tree grid there is a wider distribution of ToAs, compared to grids with large meshedness, like the Square and IEEE 118 Bus test power grids.

Plotting the ToA versus an effective distance $r_{\mathrm{eff}}$, which we introduced here in order to take into account the inhomogeneous distribution of inertia, we find a much stronger correlation with the ToA than with geodesic distance. The increase of correlation is found to be particularly strong in tree like grids. This is confirmed quantitatively by obtaining a larger Pearson correlation coefficient between ToA and $r_{\mathrm{eff}}$ than with *r*. Remarkably, a ballistic equation for the ToA with a velocity, as derived from the swing equation, provides a strict lower bound for all effective distances $r_{\mathrm{eff}}$ in all power grids. thereby yielding a reliable estimate for the smallest time a disturbance needs to propagate that distance as function of system parameters, in particular inertia.

We thereby conclude that in the analysis of contingencies of power grids it may be advisable that system designers and operators use the effective distance $r_{\mathrm{eff}}$, taking into account inhomogeneous distribution of inertia as introduced in Eq. (12), to locate a disturbance. Moreover, our results provide evidence for the importance of the network topology as quantified by the meshedness coefficient $\beta$. Further studies will be necessary to find the optimal magnitude and precise location of inertia and other control measures in real power grids to keep power grids resilient beyond the energy transition. Being aware of the results presented here, which show that a decrease of the magnitude of grid inertia increases the velocity of disturbances and reduces the time a disturbance needs to spread throughout the grid, may help to optimise the design and positioning of control devices in future power grids.
